# *IRF7*-deficient MDBK cell based on CRISPR/Cas9 technology for enhancing IBRV replication

**DOI:** 10.3389/fmicb.2024.1483527

**Published:** 2024-12-03

**Authors:** Guiyang Ge, Dongli Li, Qian Ling, Lihui Xu, Emad Beshir Ata, Xiaolin Wang, Keyan Li, Wen Hao, Qinglong Gong, Jianming Li, Kun Shi, Xue Leng, Rui Du

**Affiliations:** ^1^College of Animal Science and Technology, Jilin Agricultural University, Changchun, China; ^2^Wengniute Banner Agriculture and Animal Husbandry Bureau, Chifeng, China; ^3^College of Chinese Medicine Materials, Jilin Agricultural University, Changchun, China; ^4^Department of Parasitology and Animal Diseases, Veterinary Research Institute, National Research Centre, Giza, Egypt

**Keywords:** CRISPR/Cas9, gene knockout, *IRF7*, IBRV, viral replication

## Abstract

*Infectious bovine rhinotracheitis* (IBR), characterized by acute respiratory lesions in cattle, is a major infectious disease caused by *bovine alphaherpesvirus-1* (BoAHV-1). Control of this disease is primarily depending on vaccination. Madin-Darby bovine kidney cells (MDBK) being the main host cells and the important production platform for IBR vaccines. However, innate immune genes inhibit viral replication. Accordingly, the aim of this study was developing of *IRF7* gene deleted MDBK cells to facilitate the production of high-titer vaccines. The CRISPR/Cas9 technology was used to knock out the *IRF7* gene in MDBK cells and the impact on virus replication was examined using virus growth curves, CCK-8 assays, cell scratch assays, and qPCR. The knockout of the IRF7 gene in MDBK cells led to an increased replication capacity of IBRV and a significant reduction in type I interferons expression, specifically IFN-α and IFN-β. This indicates that *IRF7*^−/−^MDBK cell lines can effectively result in production of IBRV with high-titer, which will enhance the development of inactivated or attenuated vaccines.

## 1 Introduction

The infectious bovine rhinotracheitis (IBR) is one of the contagious respiratory diseases affecting cattle with a high significant economic impact on their production sector (Wang et al., 2023; Potgieter, 1997). It is caused by infection with the *bovine alphaherpesvirus-1 (BoAHV-1)* which has a relatively short replication cycle, and the host's immune system is unable to clear the virus in a short period. As a result, a status of latency might be evolved as the virus can persist in the host's sciatic and trigeminal nerves for extended periods of time. Reactivation of the virus in these latent carriers due to any stress factor is typically the main source of outbreaks in the cattle herds (Zhu et al., 2017).

The disease has a worldwide distribution but mainly in Europe, Latin America, Africa, and parts of Asia but with variable percentage of prevalence. Currently, the prevalence of the *BoAHV-1* infection in China remains high. A meta-analysis suggests that the prevalence is ~40% (Chen et al., 2018). Globally, only regions such as Australia, Germany, Denmark, Finland, Sweden, and the United Kingdom have reported successful eradication of the disease (Iscaro et al., 2021; Righi et al., 2023). The wide prevalence of the disease results in a significant economic losses to the global livestock industry mainly due to decrease in the milk production of cows, the reproductive performance of bulls, and the draft power of cattle (Toker and Yeşilbag, 2021).

Innate immunity is the host's first line of defense against pathogen invasion and replication (Akira et al., 2006). In this process, Interferon regulatory factor (IRF) 7 plays a crucial role. In normal cells, *IRF7* exists in an inactive monomeric form in the cytoplasm (Ma et al., 2023). Upon viral infection, the toll-like receptors (TLRs) 7 and 9 signaling cascade is activated. Consequently, it leads to the formation of a complex involving TNF receptor-associated factor 6 (TRAF6), *IRF7*, Myeloid Differentiation Primary Response Gene88 (*MyD88*), Interleukin-1 Receptor-Associated Kinase 4 (IRAK4) and Interleukin-1 Receptor-Associated Kinase 1 (IRAK1). Subsequently, *IRF7* is phosphorylated by *IRAK1* or Inhibitory Kappa B Kinase α (IKKα), leading to its dimerization and nuclear translocation. This process stimulates the expression of interferon (IFN-α/β) in response to RNA or DNA viruses (Ikushima et al., 2013; Konno et al., 2009; Ning et al., 2011). Viruses use different strategies to counteract the role of IRF7. Research indicates that Ebola virus VP35 promotes SUMOylation modification of IRF7, thereby blocking type I IFN expression (Chang et al., 2009). Additionally, viruses encode immediate early (IE) response proteins to counteract the functions of IRFs to ensure successful host infection and establish latency. The immediate early protein RTA of Kaposi's sarcoma-associated herpesvirus (KSHV), which possessing E3 ligase activity, ubiquitinates and degrades *IRF7* (Yu et al., 2005). Another IE protein, ORF45, can inhibit the phosphorylation and nuclear translocation of *IRF7* (Zhu et al., 2010, 2002). Meanwhile, herpes simplex virus can target *IRF7* through the immediate early protein ICP0 to suppress type I IFN activation (Shahnazaryan et al., 2020). When the *IRF7* gene is deleted in cells, it can significantly increase the replication of the associated virus, thereby increasing the viral titer (Akira et al., 2006; Mayuramart et al., 2022; Kim et al., 2020). These results highlight the crucial role of *IRF7* in antiviral defense. Furthermore, increasing virus replication is a crucial factor for obtaining harvest of high titer which facilitate vaccine production.

Clustered Regularly Interspaced Short Palindromic Repeats (CRISPR)/Cas9 is a revolutionary gene editing technology that has been successfully applied to edit various genes. This technology represents a reliable, efficient, simple, and rapid gene editing method and serves as the third generation of gene editing technology after zinc finger nucleases (ZFN) and transcription activator-like effector nucleases (TALEN). It has played an important role in gene function research (Li et al., 2023). Therefore, a comprehensive understanding of the replication dynamics of IBRV in cells is crucial to ensure the sustainable development of the cattle industry. In this study, the CRISPR/Cas9 technology was used to generate *IRF7*^−/−^ gene deleted MDBK cell lines. The produced *IRF7* knockout cells were used to assess its impact on IBRV replication.

## 2 Materials and methods

### 2.1 Cells and viruses

MDBK cells and HEK293T were obtained from the China Veterinary Culture Collection Center (CVCC, Beijing). While the IBRV JL5 strain was preserved in our laboratory. The cells were cultured in Dulbecco's Modified Eagle Medium (DMEM) supplemented with 10% fetal bovine serum (Gibco, USA). The cells were maintained at 37°C in a humidified incubator with 5% CO_2_.

### 2.2 Plasmids and strains

LentiCRISPRV2, psPAX2, PMD2.G vectors and DH5α receptor cells were kept in our laboratory.

### 2.3 Gene knockout site selection and sgRNA design

The coding sequence of the *IRF7* gene (NM001105040.1) was obtained from the online NCBI database website (https://www.ncbi.nlm.nih.gov/) to determine the distribution of the exonic regions. Using the online sgRNA design tool, two pairs of sgRNA sequences targeting the exonic region of the *IRF7* gene were designed and synthesized by Sangon Biotech Co., Ltd. (Changchun, China) (Table 1).

**Table 1:** Nucleotide sequence of the primers used in this study.

**Primer name**	**Sequence (5^′^-3^′^)**
sgRNA1-F	CACCGAGTCTCCGAAGAGCACGCGC
sgRNA1-R	AAAC GCGCGTGCTCTTCGGAGACT
sgRNA2-F	CACCG GGCAGGTGGCCGCTCCGCAG
sgRNA2-R	AAAC CTGCGGAGCGGCCACCTGCC
*IRF7*-F	CCTTTGCGGAGGGACCAATG
*IRF7*-R	CTTCCTCACCTGGGCACCCC
IFN-α-F	GTGAGGAAATACTTCCACAGACTCACT
IFN-α-R	TGAGGAAGAGAAGGCTCTCATGA
IFN-β-F	CCTGTCCTGATTTCATCATGA
IFN-β-R	GCAAGCTGTAGCTCCTGGAAAG
β-actin-F	CAAGGAGAAGCTCTGCTACG
β-actin-R	GATGTCGACGTCACACTTCA

The underscore represents the BsmBI restriction enzyme recognition site.

### 2.4 Construction of lentiviral vector plasmid

For construction of the Lentiviral vector plasmid, the reaction mixture was prepared in a PCR tube as follows: 1 μl of T4 ligase, 0.5 μl of T4 PNK, 1 μl from each of the forward and reverse primers (100 μmol/L) and ddH_2_O to a final volume of 10 μl. The annealing step was performed in a thermal cycler with the following thermal program including incubation at 37°C for 30 min, heating to 95°C for 5 min, and then cooling down to 25°C at a rate of 5°C per minute.

The LentiCRISPRv2 vector was digested with the restriction enzyme BsmBI. The digestion reaction was prepared in a total volume of 50 μl, consisting of 20 μl of LentiCRISPRv2, 5 μl of 10 x NEB Buffer, 2.5 μl of BsmBI, and 22.5 μl of ddH_2_O. The mixture was incubated at 55°C for 60 min. The obtained digested products were analyzed by 1% agarose gel electrophoresis, and the target bands were excised and purified using the E.Z.N.A.^®^ Gel Extraction Kit (V-spin) (OMEGA, USA). The purified, linearized LentiCRISPRv2 vector was ligated to the annealed sgRNA. The ligation reaction was carried out in a total volume of 10 μl, containing 0.5 μl of linearized LentiCRISPRv2, 1 μl of sgRNA, 1 μl of T4 DNA Ligase, 1 μl of 10x ligation buffer, and 6.5 μl of ddH_2_O. The mixture was gently mixed and incubated at 16°C for 2 h, followed by overnight incubation at 4°C. The ligated vector was then transformed into competent DH5α cells, and plasmid extraction was performed using the E.Z.N.A.^®^ Endo-Free Plasmid Extraction Kit (OMEGA, USA). The correct insertion and right orientation ‘of the sgRNA was verified by sequencing at Sangon Biotech Co., Ltd. (Changchun, China).

### 2.5 sgRNA lentivirus packaging

The 293T cells were passaged and kept at 37°C incubator with 5% CO_2_. The cell density was monitored, and transfection was performed when the cells had a 70–80% confluency. The transfection reaction mixture was prepared as follows: 3 μg LentiCRISPRv2-sgRNA1, 3 μg LentiCRISPRv2-sgRNA2, 4 μg PspAX2, 1.5 μg PMD2.G and Opti-MEM was added to a final volume of 250 μl. The mixture was incubated at room temperature for 15 min before being added slowly dropwise to the 293T cells. The cells were then incubated at 37°C in a 5% CO_2_ incubator. 16 h of post-transfection, the medium was replaced with fresh complete medium containing 10% fetal bovine serum (FBS). Viral supernatants containing sgRNA1 and sgRNA2 were collected at 48 and 72 h post-transfection. These viral supernatants were used for the subsequent generation of the gene knockout cell lines.

### 2.6 Construction of the stable *IRF7* gene knockout MDBK cell line

The resuspended MDBK cells were added to the obtained packaged virus solution with complete medium at a ratio of 1:1 by volume (V/V). Parallelly, MDBK cells without lentivirus were kept as a negative control group (mock cells). After 24 h, the lentivirus and complete medium were replaced with the same constituents supplemented with 1.0 μg/mL puromycin (Solarbio China). The cells were screened continuously for 14 d until all the cells in the negative control group were dead. Subsequently, cells were digested using 0.25% trypsin, and the cells were diluted to 1 × 10^3^ cells/mL using serum-free medium, followed by 10-fold dilution to have 1 cell per 0.1 mL of medium using 5% FBS medium. The diluted cell suspension was distributed to 96-well plates using a volume of 0.1 mL per well. After 4–5 days of incubation, wells containing multiple cells were removed, and wells with single cells and normal morphology were labeled. After 7–9 days of incubation, the selected cells were up gradually transferred to 6-well plates.

### 2.7 PCR identification of IRF7 gene knockout MDBK cell line

The resulting cell genomic DNA was extracted according to the manufacturer's instructions manual (Omega, USA), amplified using the primers listed in Table 1. The amplification system consisted of 12.5 μl 2 × Taq Plus Master Mix (Takara Japan), 0.5 μl each of *IRF7*-F and *IRF7*-R, 1 μl DNA, and 9.5 μl ddH_2_O. The amplification thermal program was performed for 30 cycles of 95°C for 3 min, 95°C for 15 s, 60°C for 15 s, and 72°C for 30 s. The obtained amplicons were extracted from the gel and sent for sequencing by Sangon Biotech Co., Ltd. (Changchun, China).

### 2.8 Western blot analysis

The proteins of *IRF7*^−/−^ MDBK and WT MDBK were extracted according to the protein extraction manual (Bestbio, China). The protein samples were quantified using the BCA assay kit (Beyotime, China). Subsequently, 5 × SDS loading buffer was added, and the samples were denatured at 100°C for 10 min. Polyacrylamide gel electrophoresis was performed, followed by transfer to a nitrocellulose membrane. The 5% skimmed milk was used as a membrane blocking buffer for 2 h followed by washing. The blocked membrane was incubated with the primary antibody (Sigma, PRS3941; 1:5,000) at 4°C on a shaker for overnight. After washing the membrane three times with TBST buffer, the membrane was incubated with secondary antibodies (Proteintech SA00001-2 1:2,000) at room temperature for 2 h. After three washing cycles with TBST, the membrane was visualized using the ECL detection kit (Vazyme, China) and imaged with a chemiluminescence imaging system (Sinsage, China). The GAPDH (Proteintech 10494-1-AP 1:5000) was utilized as a normalization endogenous protein.

### 2.9 Analysis of *IRF*7^−/−^ MDBK cell proliferation

#### 2.9.1 Cell viability

Two cell suspensions were inoculated into 96-well plates, with three replicates per group. The cells were incubated for 12, 24, 36, and 48 h. Following the incubation period, 10 μl of Cell Counting Kit-8 (CCK-8). solution was added to each well, and the incubation was continued for a further 2 h. The absorbance values of the wells at 450 nm were measured using an enzyme-labeled instrument (Bio Tek Instruments USA).

#### 2.9.2 Cell quantification

Both types of cells were inoculated into 24-well plates, and the total number of cells and the volume of culture medium were equally distributed in replicates based on the cell count. Following incubation for 0, 12, 24, 36, 48, 60, and 72 h, both cell types were digested into a cell suspension. The cell count was performed, and the mean values were calculated.

#### 2.9.3 Healing capability

Two types of cells in logarithmic growth phase were selected, and the cell concentration was adjusted to be 5 × 10^5^ cells/mL followed by inoculation into 6-well plates. After the cells attachment to the well, perpendicular scratches was done in the cells monolayer, and the floating cells were washed away with PBS, 2 mL of fresh complete medium was added, and the plates were incubated. The degree of cell confluence was observed and photographed at 0, 1, 2 and 3 days post-seeding.

### 2.10 One-step growth curve of virus IBRV JL5 strain in *IRF*7^−/−^MDBK cells

The *IRF*7^−/−^ and WT MDBK cells were separately inoculated into T25 cell culture flasks. IBRV was used for cell infection using a MOI of 0.1 and harvested at 12, 24, 36, 48, 60, 72, 84, and 96 h post inoculation. The viral fluids were 10-fold serially diluted from 10^−2^ to 10^−10^ and each dilution was inoculated into 96-well plates with 8 replicates for each of *IRF*7^−/−^ and WT MDBK cells, respectively and negative and positive controls were used simultaneously. The cytopathic effect (CPE) was observed 4 days after inoculation and the TCID_50_ was calculated using the Reed-Muench method and plotted as a viral growth curve.

### 2.11 Detection of TK and gE genes by qPCR

The two types of cells were separately inoculated into 6-well plates. Upon obtaining 75% cell confluence, the IBRV solution was inoculated at a concentration of MOI = 0.1 adding to using a negative cell control. The total RNA was extracted using Trizol reagent (TransGen Biotech, China) at 0, 12, 24, 48, and 60 h according to the manufacturer's instructions, respectively. Reverse transcription of RNA was performed according to the instructions of the reverse transcription kit (Takara, Japan), and the resulting cDNA was 20-fold diluted for qPCR template. The qPCR reaction system was consisted of 20 μl: 6 μl cDNA, 10 μl TB Green solution (Takara, Japan), 0.5 μl each of forward and reverse primers (TK and gE) (Table 1), and 3 μl ddH_2_O. The qPCR was carried out in eight tubes by fluorescence quantitative PCR in a qTOWER3 G instrument (Analytik Jena AG Jena, Germany). The Running thermal conditions were 40 cycles of 95°C for 30 s, 95°C for 10 s and 65°C for 30 s. β-actin was used as the reference gene, with three replicates per sample. The relative expression levels of each gene were calculated using the 2^−ΔΔCt^ method. On the same way, detection of IFN-α and IFN-β using the IFN-α and IFN-β primers (Table 1) was done, respectively but on the extracted RNA samples from 36 h post infection only.

### 2.12 Statistical analysis

All data were derived from three independent experiments, with each experiment conducted in triplicate. Error bars represent the standard error of the mean (SEM) across the three independent experiments. The symbol (^*^) indicates *p* < 0.05. Statistical data were processed by one-way analysis of variance in GraphPad Prism 7.0 software (GraphPad Prism, USA).

## 3 Results

### 3.1 Lentiviral plasmid construction

**Figure 1:**
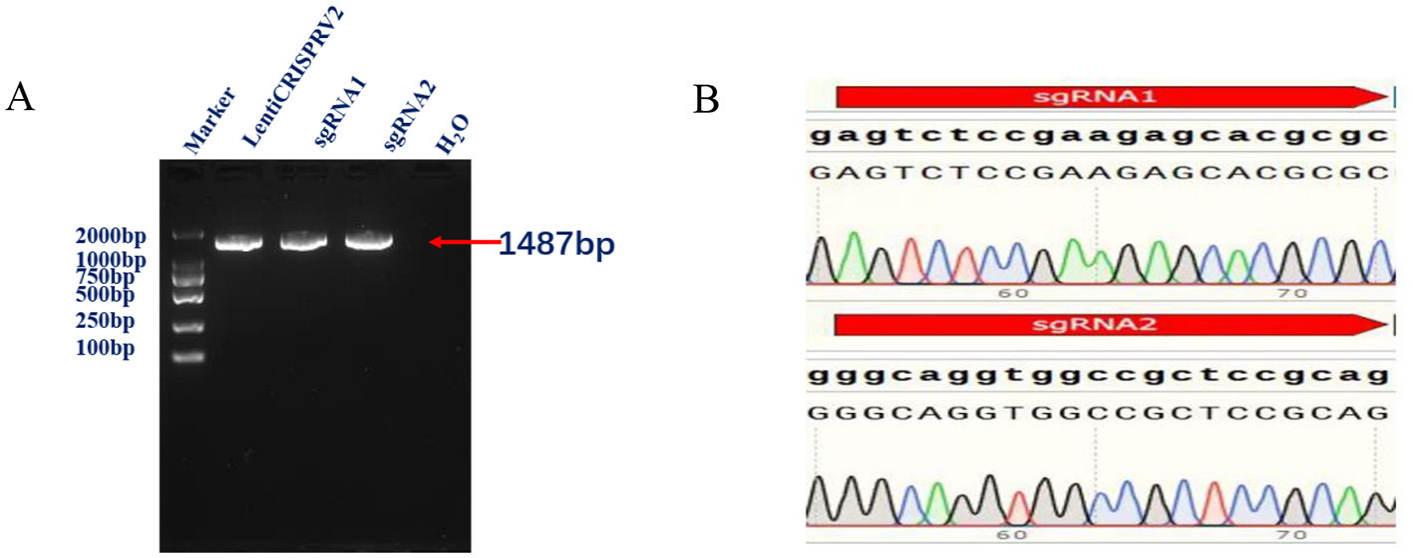
**(A)** Conventional PCR identification results shows amplification of 1,487 bp representing successful lentiviral plasmid construction. **(B)** Validation of the constructed CRISPR/Cas9 vector through Sanger sequencing shows presence of a 17 base pair deletion of the developed *IRF7*^−/−^ cells comparing to the wild type.

The results of the polymerase chain reaction (PCR) electrophoresis demonstrated the presence of a band of approximately 1,487 bp, which is consistent with the expected results (Figure 1A). The sequencing results demonstrated that both sgRNA1 and sgRNA2 were successfully ligated to sgRNA within the pLentiCRISPRv2 vector (Figure 1B).

### 3.2 Identification of *IRF7* knockout MDBK cell line

**Figure 2:**
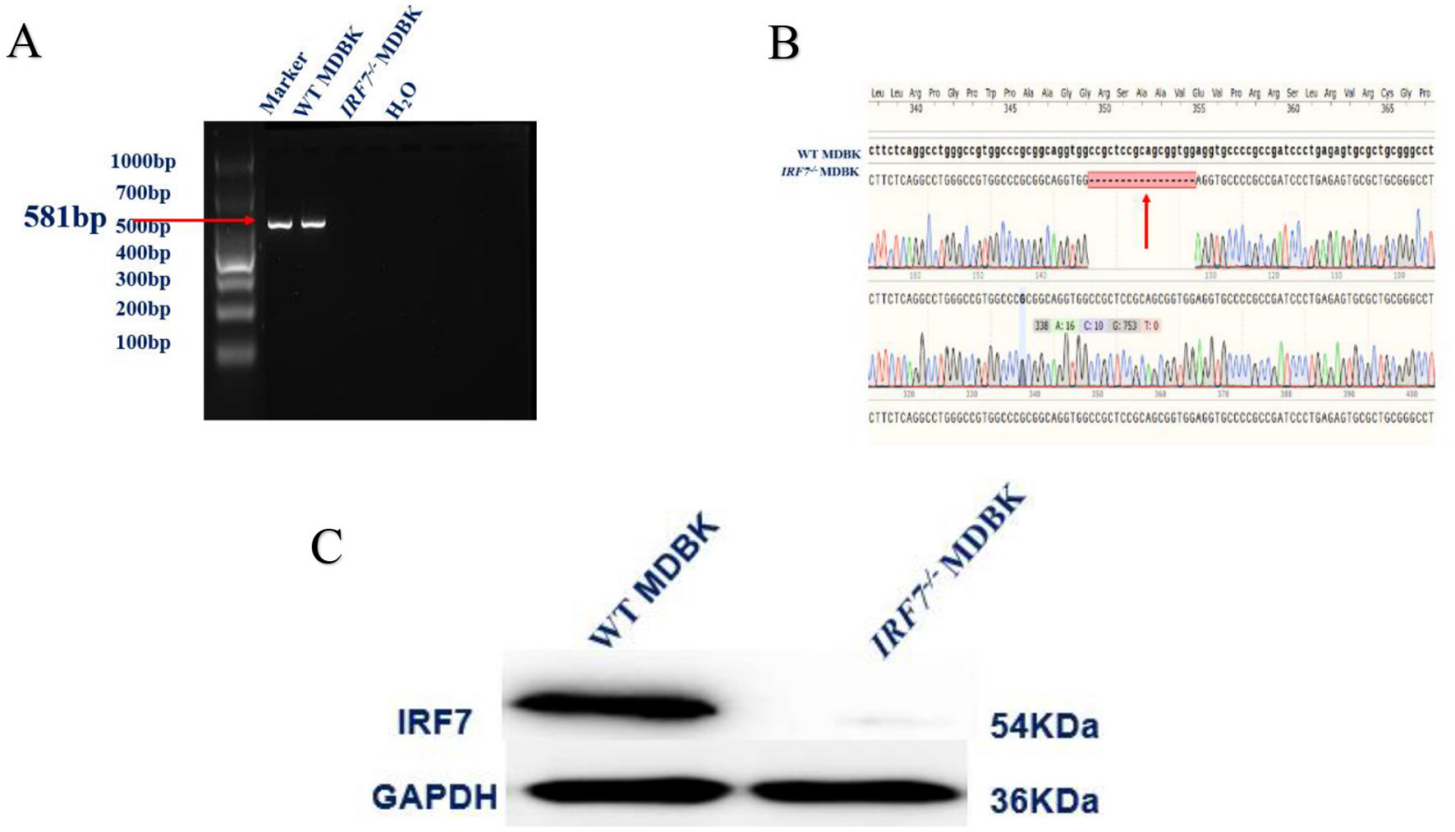
**(A)** Conventional PCR identification results. **(B)** Sanger sequencing results. The red arrow indicates the deletion site. **(C)** Western blot detection of IRF7 protein expression.

After lentiviral infection and puromycin screening of MDBK cells, an *IRF7*^−/−^ MDBK cell line was successfully obtained, which was sequenced and showed the presence of a 17 base pair deletion (Figures 2A, B). Western blot validation demonstrated the absence of the *IRF7* protein in the knockout cell group, confirming the successful generation of the *IRF7* knockout cell line (Figure 2C).

### 3.3 Cell viability, quantification and healing capability assay

**Figure 3:**
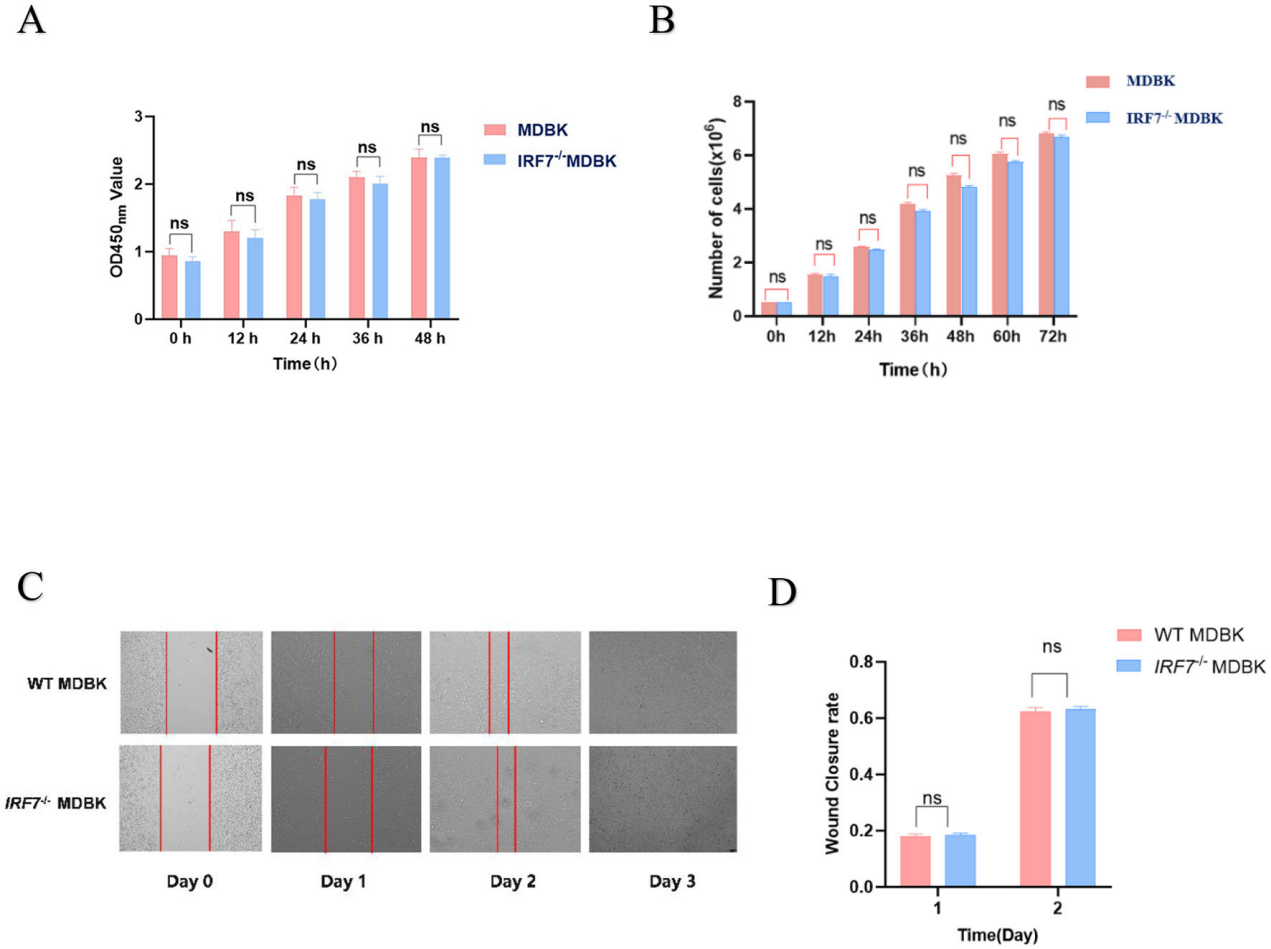
**(A)** CCK-8 assay: evaluation of the vitality of both cell types. **(B)** Quantitative analysis of cell count at different time points. **(C)** Cell scratch assay: healing progress of cells within 4 days after creating a scratch. **(D)** Image J analysis: quantitative assessment of the cell scratch test using Image J, where 1 and 2 represent the healing rates on days 1 and 2, respectively, relative to day 0.

The viability of the two cell types was detected by CCK-8, and the results showed that there was no significant difference in the viability of the two cells across the time (*P* > 0.05) (Figure 3A), and the results of the counting of the two cells showed that there was no significant difference in the number of cells between the two times with the change of time (Figure 3B) (*P* > 0.05), which indicated that the knockdown of IRF7 had no significant effect on the growth of the MDBK cell rate.

To further assess the effect of IFR7 gene deletion on cell migration activity, a cell scratch assay was performed. Statistical analysis of the results obtained at the 0, 1, 2, and 3 d showed no significant difference in the healing rate of the two tested cell (*P* > 0.05), and both types of them were completely healed by the third day (Figures 3C, D).

### 3.4 Knockout of *IRF7* increased viral titers

**Figure 4:**
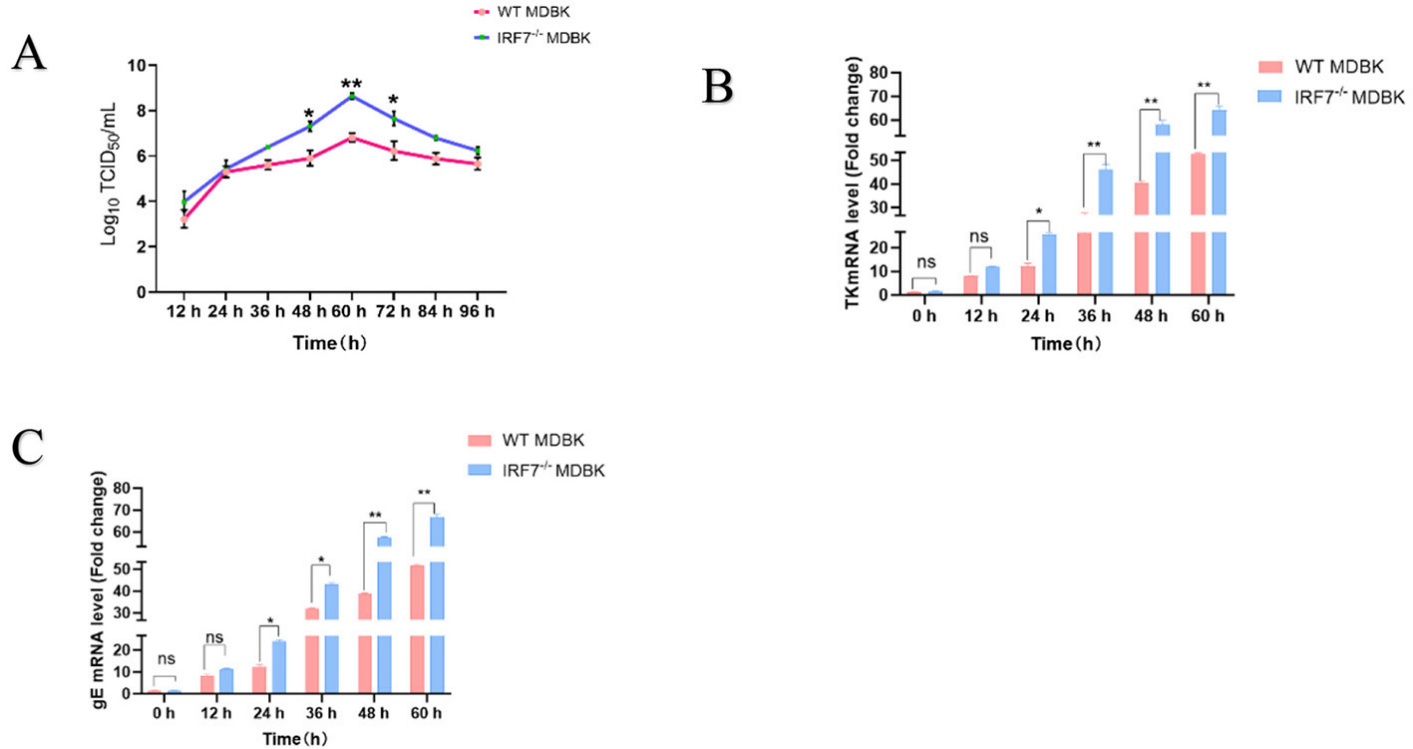
**(A)** One-step growth curves of virus infections at the WT MDBK and *IRF*7^−/−^ MDBK cell lines. **(B)** TK genes detection of IBRV JL5. **(C)** gE genes detection of IBRV JL5. *P < 0.05;**P < 0.01.

The results of viral growth curves of IBRV JL5 infected *IRF7*^−/−^ MDBK and the normal (wild type) MDBK cells showed that viral replication was accelerated, and the virus was in logarithmic growth phase at 36–60 h. The viral growth curve of IBRV JL5 infected *IRF7*^−/−^ MDBK cells was significantly higher than that of normal MDBK cells (*P* < 0.01). At 60 h the viral titer reached the highest level in the *IRF7*^−/−^ MDBK group which was significantly higher than that of the WT MDBK cell-infected group (*P* < 0.01), suggesting that *IRF7* gene deletion promotes the proliferation of IBRV JL5 strain on MDBK cells (Figure 4A).

Fluorescence quantitative PCR was employed to assess the expression of IBRV TK and gE genes in both cell types. From 24 h onwards, MDBK *IRF7*^−/−^ cells exhibited significantly higher mRNA expression of TK and gE compared to the WT MDBK cells (Figures 4B, C). In conclusion, the deletion of IRF7 in MDBK cells can significantly enhance the replication ability of IBRV.

### 3.5 Knockout of IRF7 inhibited expression of type I IFN

**Figure 5:**
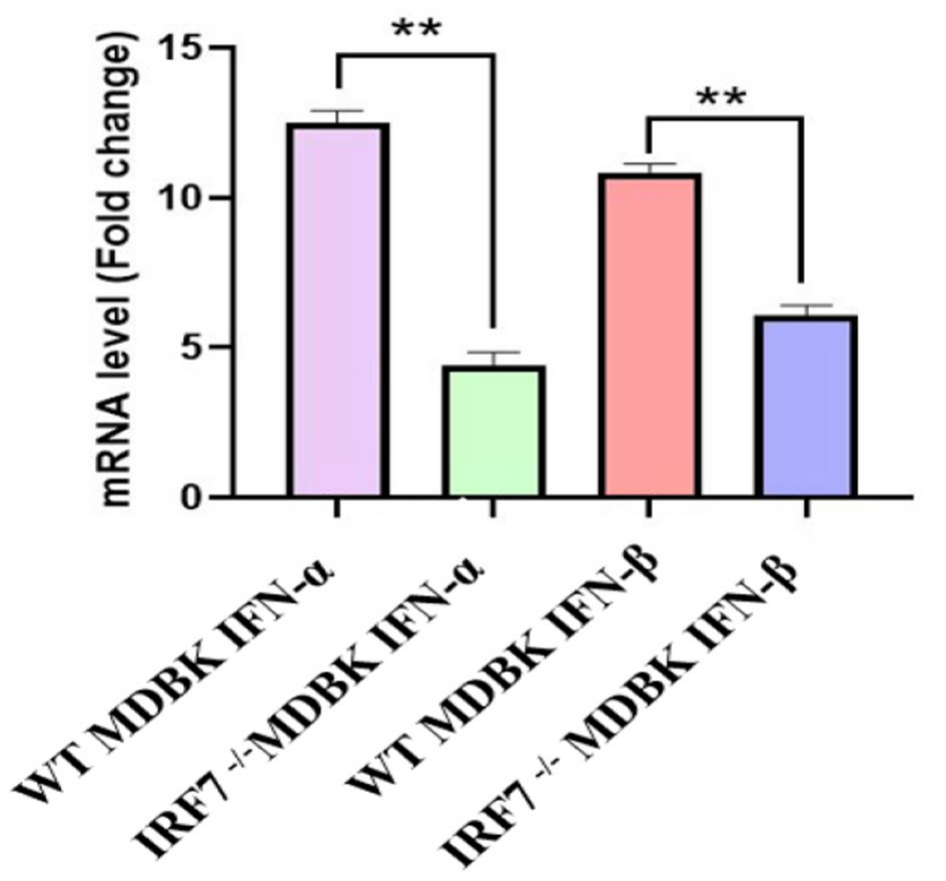
Effect of *IRF7* deletion on the expression of IFN-α and IFN-β. Expression levels of IFN-α and IFN-β 36 h after MOI = 0.1 IBRV infection in both cell types. **P < 0.01.

For determination of the IRF7 deletion on the expression of the different interferons. Two types of cells were stimulated with IBRV JL5. Subsequently, qPCR was used to measure mRNA levels of IFN-β and IFN-α. IFN-α and IFN-β which are the two most common and studied isoforms of type I interferons, and playing crucial roles in antiviral responses and immunomodulation. IFN-α primarily combats viral infections by activating ISGs, while IFN-β regulates immune cells and inflammatory responses. The results demonstrated that the expression levels of IFN-α and IFN-β were significantly higher in MDBK cells compared to *IRF7*^−/−^MDBK cells (*P* < 0.05) (Figure 5).

## 4 Discussion

IBRV was firstly identified in the United States in the 1950s and has since been progressively detected in cattle herds across Europe, Americas, Asia and Africa (Iscaro et al., 2021; Kipyego et al., 2020; Valas et al., 2023). In 1980, the virus was discovered in imported cattle from New Zealand (Xu et al., 2017). In recent years, with the rapid development of the cattle industry in China, especially in dairy farming, the frequent import and inter-regional transportation of cattle have increased the threat of IBRV spreading. However, we currently lack effective means to control IBRV infection. Additionally, when the animal body is infected with a virus, the immune system mainly acts through two mechanisms to counteract viral effects, including innate immunity and acquired immune responses (Zhou et al., 2021). Innate immunity serves as the first line of defense in the body's resistance against viral infection. To cope the infection of the various pathogens, host cells express and release different types of IFN which induces the production of hundreds of interferon-stimulated genes (ISGs) through cell surface IFN receptors, thereby enhancing the host's antiviral capabilities (de Veer et al., 2001). As a key member of the interferon regulatory factor (IRF) protein family, *IRF7* plays a critical role in the innate immune system through responding to downstream pathogens and recognize receptor infections. It serves as a “primary” regulator in the production of IFN, which plays an important role in innate immunity (Honda et al., 2005). In addition, preliminary research from our laboratory indicates that the transcription level of the *IRF7* gene is significantly upregulated in MDBK cells following IBRV infection (unpublished). Therefore, to investigate the impact of the *IRF7* gene on IBRV replication, a cell line with a deleted *IRF7* gene was generated. Furthermore, we conducted an analysis of the consequences of *IRF7* deficiency on IBRV proliferation, providing valuable insights for the development of IBRV vaccines.

In the past decade, there has been a significant increase in the number of publications related to CRISPR/Cas9 and gene editing in the fields of life sciences and virology (Teng et al., 2021). It has been widely used in research at both the cellular and organismal levels in animals (Gandhi et al., 2017; Torres-Ruiz and Rodriguez-Perales, 2017). This gene editing technology, in contrast to transient siRNA knockdown, can be used to generate stable modified cell lines, providing more accurate information for research and more opportunities to explore gene function. Therefore, CRISPR/Cas9 technology was used to construct the MDBK *IRF7* knockout cell line and ensured the viability of these cells through validation (Figures 2, 3). Western blot analysis confirmed the absence of IRF7 protein expression in the knockout cell line, validating the successful establishment of *IRF7*^−/−^ MDBK cell lines. By comparing the growth performance of the two cell lines, no significant differences were observed between them (Figure 3). This result confirms successful construction of the MDBK *IRF*7^−/–^ cell lines. Although numerous studies indicated that the efficiency of Cas9/gRNA-mediated gene knockout in mammals is around 40–80%, the type of cells and the maturity of the technology remain important factors affecting knockout efficiency (Zhang et al., 2017). In this study, *IRF7* gene knockout in MDBK cells was achieved for the first time, demonstrating that Cas9/gRNA technology is effective for gene editing in bovine cell lines. This breakthrough provides a valuable resource for further investigation into gene functions.

The tolerance of cell lines to viral infection depends on the interaction of multiple host factors (Komissarov et al., 2022). Currently, there have been numerous studies reported on the replication of IBRV. In MDBK cells infected with BoAHV-1, all three major MAPK pathways are activated to counter viral infection; however, only the JNK signaling pathway is uniquely essential for virus replication (Zhu et al., 2016). In addition, research has shown that DNA damage-inducible transcript 3 (DDIT3) counteracts the innate immune response through the DDIT3-SQSTM1-STING pathway and promotes BoAHV-1 replication (Wang et al., 2022). These research findings demonstrated that the impact of cells on IBRV replication may occur through multiple pathways. In our further studies, we discovered that the *IRF7* gene also plays a crucial role in the antiviral process (Zhou et al., 2021, 2012; Zhong et al., 2023; Zhao et al., 2021; Ren et al., 2020). However, there is limited research on MDBK cells and IBRV virus. Therefore, this study investigated the replication of IBRV JL5 strain in the constructed *IRF7*^−/−^MDBK cell lines. The results showed a significant increase in the TCID_50_ of the virus in the *IRF7*^−/−^MDBK cell lines comparing to the wild type. The qPCR analysis of TK and gE mRNA expression revealed much higher levels in the *IRF7*^−/−^MDBK cell lines compared to MDBK cells (Figure 3B). This suggests that the loss of *IRF7* enhances the replication capability of IBRV.

IFNs were first discovered in the 1950s. They constitute a class of biologically active glycoproteins secreted by various cells (Isaacs et al., 1957). They are produced by host cells and exert a broad-spectrum antiviral, antitumor, and immunomodulatory roles (Aric and Belardelli, 2012; Minayoshi et al., 2018). Interferons (IFNs) can be classified into three types based on genetic characteristics, receptor specificity, and chromosomal location. Type I IFNs include more than 20 subtypes, such as IFN-α and IFN-β, with primary functions of antiviral and antitumor activity (Wang and Fish, 2019). IFN induces the production of interferon-stimulated genes (ISGs) to exert its antiviral effects, with different ISGs responsible for inhibiting infections by different types of viruses (Schneider et al., 2014). *IRF7* can influence the expression of Type I IFN through various pathways including formation of a complex with MyD88 in the cytoplasm, thereby activating the expression of IFN-α and IFN-β (Honda et al., 2004). In the cascade of antiviral immune responses, the translocation of *IRF7* from the cytoplasm to the nucleus requires phosphorylation. Phosphatase 1 interacts with *IRF7* and dephosphorylates it, significantly diminishing the activity of *IRF7* and impeding the production of IFN-α mediated by *IRF7* (Zhao et al., 2021). In some other studies of herpesviruses such as Epstein-Barr virus (EBV) and Marek's disease virus (MDV), it has been found that they also inhibite the expression of type I IFN through IRF7 (Wang and Fish, 2019; Gao et al., 2019). In this study, we also found that the expression of IFN-α and IFN-β was suppressed after *IRF7* gene was knocked down.

## 5 Conclusion

In this study, *IRF7*^−/−^ MDBK cell line was developed. The absence of the IRF7 gene resulted in reduced expression of Type I interferons, which in turn enhanced the replication of IBRV. The *IRF7*^−/−^MDBK cell line can be used to investigate the virus pathogenesis. Additionally, it provides a new solution for optimizing the production process of bovine infectious rhinotracheitis vaccines. By exploiting the effect of *IRF7* gene deficiency in MDBK cells on the proliferation of bovine infectious rhinotracheitis virus, the virus production capacity can be significantly increased.

## Data Availability

The original contributions presented in the study are included in the article/Supplementary material, further inquiries can be directed to the corresponding author.
