# Supplementary material for: IRF7-deficient MDBK cell based on CRISPR/Cas9 technology for enhancing IBRV replication
Source: Front Microbiol. 2024 Dec 3;15:1483527. doi: 10.3389/fmicb.2024.1483527 (PMC11649632; doi:10.3389/fmicb.2024.1483527)
Supplement: Supplementary file 1 [file Image_1.pdf]

## The construction process diagram for the *IRF7*$^{-/-}$ MDBK cell line.

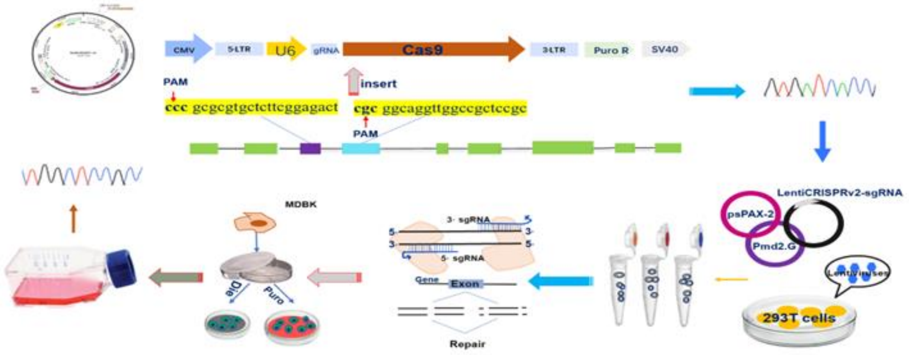


**Fig.S1** Process diagram for constructing *IRF7*$^{-/-}$ MDBK cell lines

Two sgRNAs were designed based on the exon regions cng. Lentivirus packaging was per formed in 293T cells, and the generated lentivirus was used to transfect MDBK cells usin g the CRISPR/Cas9 technology, leading to the selection of *IRF7* gene knockout cell line.
